# A novel nucleotide insertion in S gene of hepatitis B virus in a chronic carrier

**DOI:** 10.1186/1743-422X-7-104

**Published:** 2010-05-22

**Authors:** Wenbin Tong, Li Sun, Jilan He, Shusen He, Fei Du

**Affiliations:** 1 Institute of Microbiological Detection, Sichuan Center for Disease Control and Prevention, Chengdu 610041, PR China

## Abstract

Hepatitis B virus DNA was extracted from serum of a chronic carrier and polymerase chain reaction was performed on S gene. Direct sequencing showed a variant HBsAg with additional 4-amino acid insertion, and clone sequencing confirmed the mixture of variant HBsAg and wildtype HBsAg. Of 16 clones with 12-nucleotide insertion, 15 clones had identical AGAACAACACAA insertion between nucleotide 494 and nucleotide 495, and one clone had GGAACAACTCAA insertion in the same position plus 3-nucleotide deletion from nucleotide 491 to nucleotide 493. S114T, C121Y, T126S/A, Q129K, G130R, T131N, M133T, G145R, N146D substitution and premature stop codon were also found in those clones. However, the origin of HBV with 4-amino acid insertion in HBsAg was unclear.

## Introduction

Hepatitis B virus (HBV) infection is a global health problem. It was estimated that more than 2 billion of the global population had been infected. Of these, approximately 360 million were chronically infected and estimated 500 000-700 000 people died from complications of infection each year worldwide [1]. HBV infection was highly endemic in China, a national serosurvey conducted in 2006 showed 7.2% surface antigen(HBsAg) prevalence in population aged 1~59 years old [2]. Control and therapy of HBV-related diseases weighed great burden on both government and infected individual [3]. HBsAg, a main serological marker for diagnosis of HBV infection, comprises 226 amino acids(aa), and contains a highly conformational epitope cluster in major hydrophilic region(MHR) extending from aa99-aa169, where common α determinant is located at aa124-aa147 [4-6]. Antibodies induced by natural infection or by administration of vaccine direct against α determinant and confer cross-protective immunity to all subtype [7-9]. However, aa substitution, insertion or deletion in MHR may cause the loss of conformational epitope and altered antigenicity of HBsAg, renders HBsAg undetectable, or induces immune escape variants to evade virus clearance [10-17]. In this study we reported a novel 12-nucleotide(nt) insertion between nt494 and nt495 in S gene, which led 4-aa insertion between aa113 and aa114 compared with wildtype HBsAg.

## Materials and methods

### Sample selection and serological assay

HBV chronic carriers were identified in 2 counties(districts) of Sichuan province by serological assay and epidemiological investigation in a subnational sampling serosurvey. Partial sequence analysis of S gene was carried out for total 50 sera [18]. The serum 06A26 was selected in this study because it presented aa insertion in MHR by partial S gene sequencing.

Blood sample was drawn from a 77-year-old male patient. Serological assays were positive for HBsAg, HBeAg and anti-HBc, negative for anti-HBs and anti-HBe with available commercial ELISA kit(Wantai, China).

### PCR and sequencing

HBV DNA was extracted from serum with QIAamp DNA Mini Kit(QIAGEN, Germany) according to manufacturer's instructions. Primer pair(S006-sense, 5'-CCTGCTGGTGGCTCCAGTTC-3', postion 56-75; S007- antisense, 5'-CCAATTACATATCCCATGAA-3', position 893-874) were used to amplify complete fragment of S gene with Taq PCR Master Mix Kit(QIAGEN, Germany) [14]. PCR was performed for 40 cycles, each cycle included denaturation at 94°C for 30 sec, annealing at 55°C for 1 min, and extension at 68°C for 2 min, with initial denaturing step at 94°C for 3 min and final amplification step at 68°C for 10 min. PCR amplicon was purified with Wizard SV Gel and PCR Clean-up System(Promega, USA) according to manufacturer's instructions. Cycle sequencing reaction was performed bidirectionally with same S006 and S007 primers using Bigdye Terminator v3.1 Cycle Sequencing Kit(Applied Biosystems, USA). After purification with Sephadex G-50 Fine (Amersham, Sweden), sequencing products were analyzed on ABI PRISM 3130 Genetic Analyzer(Applied Biosystems, Japan).

PCR Amplicon of 06A26 was also cloned into PMD18-T Vector(TakaRa, China), transformed into *E. coli *Electro-Cell DH5α(TakaRa, China) and then sequenced.

S gene sequence of 06A26 PCR amplicon and 20 clones were deposited in GenBank under accession number GU145104 and GU358454-GU358473, respectively.

### Quantitation of serum HBV DNA

The amount of serum HBV DNA was measured 1.6 × 10^5 ^copies/mL on the basis of calculation from standard curve with commercial quantitative realtime PCR kit(Da'an, China) on 7300 Real Time PCR System(Applied Biosystems, Singapore).

## Results

### Nucleotide analysis of S gene

**Figure 1:**
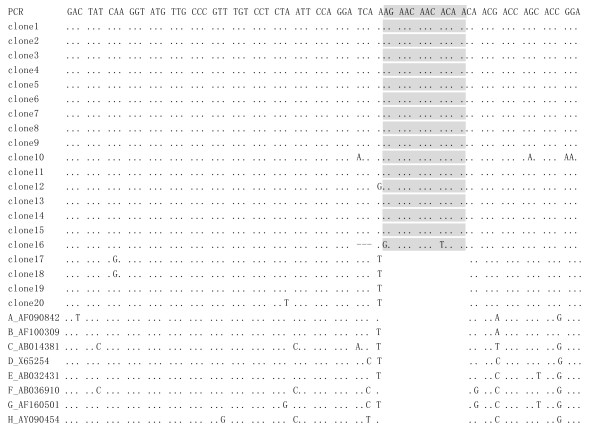
**Alignment of nucleotide sequences in the HBs1 region of 06A26 PCR amplicon and clones with genotype A-H reference isolates**. Homologous nucleotides were indicated with dots, deleted nucleotides with dashes. Heterologous nucleotides are specified. Inserted nucleotides were indicated with grey boxes.

Alignment of nt sequences in S gene of 06A26 PCR amplicon and 20 clones with genotype A-H reference isolates showed a 12-nt insertion between nt494 and nt495 in PCR amplicon and clone1-clon16. Of 16 clones with 12-nt insertion, 15 clones showed identical insertion sequence, AGAACAACACAA. Another clone showed otherwise insertion sequence, GGAACAACTCAA, and contained a 3-nt(TCA) deletion from nt491 to nt493(figure 1). Phylogenetic analysis by Mega3.1 software indicated they belonged to genotype B.

### Amino acid sequence analysis of HBsAg

aa sequences were deduced from S gene DNA sequences of 20 clones. Lys^122^, Lys^160 ^and Pro^127 ^indicated they belonged to subtype adw2.

**Figure 2:**
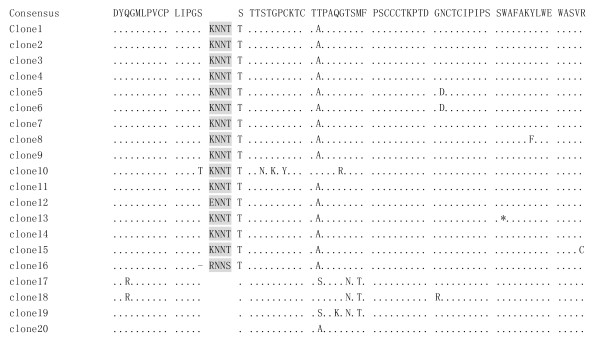
**Alignment deduced amino acid sequences in MHR(aa99-aa169) of 20 clones with consensus sequence from published Chinese isolates with genotype B/subtype adw2(GenBank accession number AB033554, AF100309, D00329, AF397207, EU305547, EU589335 and GU145102)**. One-letter code was used for amino acid in consensus sequence, homologous amino acids were indicated with dots, deleted amino acid with dash, and premature stop codon with asterisk. Heterologous amino acids were specified. Inserted amino acids were indicated with grey boxes.

Compared with consensus sequence of Chinese isolates retrieved from GenBank with same genotype/subtype, clone1-clone11 and clone13-clone15 had KNNT insertion, clone12 ENNT insertion and clone16 RNNS insertion between aa113 and aa114, respectively. In addition, clone16 contained a deletion at aa113. N146D substitution(AAC→GAC) occurred in clone5-clon6 at aa146, leading to loss of N-linked glycosylation site at this position. S114T substitution(TCA→ACA) occurred in all clones with 4-aa insertion. This 4-aa insertion alone or plus following aa114 substitution might form a possible N-linked glycosylation site. Of 4 clones with wildtype HBsAg, T131N(ACC→AAC) and M133T(ATG→ACG) substitution occurred in clon17-clone19, also forming a possible N-Linked glycosylation site(figure 2).

C121Y(TGC→TAC), T126S/T126A(ACT→TCT or ACT→GCT), Q129K(CAA→AAA), G130R(GGA→AGA), G145R(GGA→AGA) and other aa substitution compared with consensus sequence in MHR were also showed in figure 2. Premature stop codon occurred in clone13 at aa156 (TGG→TAG), aa191 (TGG→TGA) and aa196(TGG→TGA), in clone14 at aa206 (TAC→TAG) and in clone15 at aa223 (TGG→TGA), respectively.

## Discussion

Carman proposed to separate MHR into functional areas HBs1-HBs5 [19], which relate to antigenic effect of variants and their selective pressures. Since Yamamoto first reported 8-aa insertion between aa123 and aa124 in S gene [14], aa insertion/deletion in HBsAg had been reported in following studies, they included 2-aa insertion between aa122 and aa123 [15,16], 3-aa insertion between aa123 and aa124 [16], 1-aa insertion between aa121 and aa122 [20], 3-aa insertion between aa118 and aa119 [18], 2-aa deletion from aa110 to aa111 [17], 4-aa deletion from aa119 to aa122 [17], 3-aa deletion from aa113 to aa115 [21]. Those insertion/deletion were located into HBs1-HBs2, so both HBs1 and HBs2 were hot spots for aa insertion/deletion [5,16,17]. Recently, Zhou reported 5-aa insertion between aa128 and aa129 [22], which located into HBs3(also in first loop of α determinant). As sequencing data accumulate, more aa insertion/deletion in HBsAg may be found.

HBsAg was detectable in serum of the patient in this study, despite 4-aa insertion between aa113 and aa114, compared with HBsAg negative sera described previously [14,16,17,22]. Of note, HBsAg with 1-aa insertion behind aa113 in a serum was reported to be detectable only at low dilution(5 times), while HBsAg without aa insertion in other sera was detectable at high dilution(at least 16 000 times) [23]. So PCR amplicon was cloned to find co-existence of wildtype HBsAg that might contribute to HBsAg detection. Clone sequencing confirmed the mixture of variant HBsAg and wildtype HBsAg. High level of serum DNA viral load(>1 × 10^5 ^copies/mL) and 20% of wildtype HBsAg ensured HBsAg detection [16,24]. Other experiment such as site-directed mutangenesis is needed to confirm whether this 4-aa insertion alone or plus following aa114 substitution will alter HBsAg antigenicity and affect the binding capacity of anti-HBsAg [25,26].

Due to overlapping of S gene with P gene, variation in S gene may affect catalytic activity of reverse transcriptase(rt) domain in P gene and vice versa [27]. Deduced rt sequences were also analyzed. 12-nt insertion caused 4-aa insertion(KEQH, REQH, KEQL, respectively) between rt121 and rt122 in clone1-clone16, and another deletion at rt121 in clone16. Those insertion/deletion were located in junction between conserved region A and B [28]. No premature stop codon occurred in corresponding rt domain. Some reported drug-resistant mutants, such as rtF166L and rtP177L [28], were found in clone18 and clone15, respectively, and rtD205N mutant(creating premature stop codon at aa196 in overlapping HBsAg) in clone13 led to change from YMDD motif to YMND motif. However, high level of HBV DNA rival load suggested this 4-aa insertion in rt domain was less likely to be strong suppression of viral replication [11,29].

PCR and direct sequencing on PreS1/S2 gene were also performed, no insertion/deletion was found [14].

The origin of variant HBsAg was unclear. The patient had no history of vaccination, therapy with interferon-α/nucleoside analogues, administration of hepatitis B immune globulin and blood transfusion. Knowing of his infection status more than thirty years, the patient took traditional Chinese medicine available for a long time. Traditional Chinese medicine has been proven economical and efficient in liver protection and treatment, and has less side effects compared with interferon α and lamivudine [30,31]. However, "Medicine is partly poisonous", according to traditional Chinese medicine theory, they may have unknown side effects, due to complex ingredients usually used in the formulas. Though anti-HBsAg was negative in the patient serum, it might be existent under detection limit for applied method. If more sensitive methods were applied, anti-HBsAg could been detected in nearly all patients with chronic hepatitis B infection [32,33]. It was possibility that the patient first infected wildtype HBV, host immunity responded to HBV infection but failed to clear virus. Selective pressure from host immunity, traditional Chinese medicine and other factors in combination favored the replication of variant HBV with 4-aa insertion in HBsAg and outnumbered wildtype HBV.

In summary, we reported a novel 12-nt insertion in S gene of HBV, which led to 4-aa insertion in HBsAg between aa113 and aa114, but the origin of the variant HBV was unclear. A well-documented immune escape mutant G145R in one clone, might occur naturally due to lack of proof-reading activity of rt domain and exist as quasi-species viral population [34,35].

## Competing interests

The authors declare that they have no competing interests.

## Authors' contributions

WBT carried out most part of the study and drafted the manuscript. LS, JLH and SSH took part in serological detection. FD was responsible for epidemiological data analysis. All authors read and approved the final manuscript.
